# The Illinois Bill to Regulate the Practice

**Published:** 1898-12

**Authors:** 


### Abstracts and Selections.

### The Illinois Bill to Regulate the Practice.

A valued subscriber who is opposed to the bill to regulate the practice in Texas, drafted by the State Medical Association, sends us a copy of the bill now pending, we believe, before the Illinois Legislature, and requests us to publish it.

The bill reads as follows:

"A BILL

"For an Act to establish a State Board of Medical Examiners, prescribing its powers and duties, to provide for the licensing of practitioners of medicine and midwifery, and to regulate the practice of medicine and midwifery in the State of Illinois, and imposing penalties and to repeal all acts or parts of acts in conflict therewith:

"SECTION 1. Be it enacted by the People of the State of Illinois represented in the General Assembly: That a board of examiners to be known as the "State Board of Medical Examiners" shall be appointed as follows: Within sixty days after this act shall take effect, the Governor shall appoint seven (7) physicians of recognized professional ability and standing, who have received the degree of Doctor of Medicine from some recognized college in good standing with the State Board of Health, as members of said Board, no one school of practice to have a majority, who shall hold their office until their successors are appointed and qualified. The members of said Board so first appointed, shall determine by lot, their respective terms of office, so that the term of one member shall expire on December 31st, 1899, and the term of one member on December 31st in each year thereafter. And at the expiration of the term of office of each of the members of the Board, the Governor shall appoint his or their successor or successors, whose term of office shall be for the period of seven (7) years; and all vacancies occurring in the membership of the Board, by resignation or otherwise, shall be filled by the Governor, and all appointments on said Board shall be made from physicians having the qualifications prescribed herein for members of the first Board; provided, however, that all appointments shall be made with the advice and consent of the Senate, but appointments made when the Senate is not in session may be confirmed at the next ensuing session thereof.

"SEC. 2. The said Board shall, within thirty days after its appointment, meet and organize by electing a president, vice-president and treasurer from among its members, and a secretary who shall be a legally qualified physician, not a member of the Board. Such officers shall hold office until the next annual meeting of the Board, or until their successors are elected and qualified. The Board shall prescribe the duties of its officers, and shall require the treasurer to give a satisfactory bond. The Board shall adopt and have a common seal, and the president or presiding member and the secretary shall be empowered to administer oaths in taking testimony, or in any matter pertaining to the duties of the Board. A majority of the Board shall constitute a quorum for the transaction of any business. It shall adopt such rules, regulations and by-laws not inconsistent with the laws of this State and of the United States, as it may deem necessary or proper, to enable it to properly perform its duties and transact its business.

"SEC. 3. The annual meeting of the Board for the election of officers shall be held in the State Capitol on the second Tuesday of January, in each year. The Board shall also hold regular meetings on the second Tuesdays in April, July and October in each year, at such places as it may determine, and may hold other meetings at such times and places as it may deem necessary, or its duties may require. It shall keep an official record of all its proceedings and a complete file of all applications for licenses, together with the correspondence pertaining thereto, and of all examination papers, and shall make a report of its transactions, annually, to the Governor, which shall include a statement of all moneys received and disbursed by it, and an official list of all physicians and midwives authorized to practice in this State.

"SEC. 4. No person shall hereafter begin the practice of medicine or any of the branches thereof, in this State, without first applying for and obtaining a license from said Board so to do. Applications shall be in writing, and in the form prescribed by the Board, and shall be accompanied by the examination fees hereinafter specified, and with proof that the applicant is of good moral character and has received a diploma conferring upon the applicant the degree of Doctor of Medicine from some legally incorporated medical institution in good standing, as may be determined by the Board, at the time of issuing said diploma. When the application and diploma aforesaid have been inspected by the Board, and found to comply with the foregoing provisions, the Board shall notify the applicant to appear before it for examination, at the time and place mentioned in such notice. Such examination shall be in the English language, and embrace those general subjects and topics, a knowledge of which is commonly and generally required of candidates for the degree of Doctor of Medicine by reputable medical colleges in the United States. No person shall hereafter begin the practice of midwifery in this State without first applying for and obtaining a license from said Board so to do. Applications shall be in writing, and in the form prescribed by the Board, and shall be accompanied by examination fee hereinafter specified and with proof that applicant is of good moral character, and has received a diploma from a school of midwifery, complying with the requirements of the Board. When the application and proofs aforesaid have been inspected by the Board, and found to comply with the foregoing provisions, the Board shall notify the applicant to appear before it for examination, at the time and place mentioned in such notice. Such examination shall be in the English language, and of such character as to determine the qualifications of the applicant to practice midwifery. All examinations provided for in this act, shall be conducted under rules and regulations prescribed by the Board, which shall provide for a fair and wholly impartial method of examination. If the applicant successfully passes his or her examination, the Board shall issue to such applicant a license authorizing him or her to practice medicine or midwifery, as the case may be, in this State. Such license shall be in such form as may be determined by the Board, and in accordance with the provisions of this act: Provided, however, any willful violation, on the part of an applicant, of any of the rules and regulations of the Board governing examination, shall be sufficient cause for the Board to refuse to issue a license to such applicant.

"Sec. 5. The examination fee for those desiring to begin the practice of medicine under the foregoing section shall be $20.00, and for those desiring to begin the practice of midwifery the examination fee shall be $15.00, and in all cases the examination fee must accompany the application.

"Sec. 6. That all persons who, at the date of taking effect of this act, are qualified to practice medicine or midwifery, under the laws of this State, in force at the time of the passage of this act, and who desire to continue in practice, shall, within six months after the appointment and organization of the Board of Examiners, provided for in this act, apply to such Board for a license, which shall be issued by the Board on payment of a fee not to exceed fifty cents. Provided, the Board may refuse to issue such license to any such applicant for cause as hereinafter provided. All licenses issued under the provisions of this and the foregoing sections shall expire on a date to be named therein, not more than one year from the date of issue, so that all licenses outstanding at one time shall, as nearly as may be, expire on the same date.

"Sec. 7. That all licenses issued under the provisions of this act, at the date of their expiration, be renewed for a period of one year, upon payment to the Board of a renewal fee not to exceed fifty cents, which renewal shall be evidenced by a new license: Provided, however, the Board may refuse such license for cause, as hereinafter provided.

"The failure of a licentiate to obtain a renewal of license at the date of its expiration shall render such practitioner liable to the penalities provided for by this act for practicing without a license, but will not prevent application for a renewal license at some subsequent date, not later than one year from the date of expiration of his former license, within which time, subject to all the conditions for renewals heretofore provided for, the Board may issue a renewal license, which shall expire at the same date as if issued at the date of expiration of his former license. Provided, however, retirement from practice or removal from the State shall not deprive the licentiate of a right to a renewal within five years after such retirement or removal. All original licenses issued by the Board shall be signed by all the members of the Board. All renewals shall be signed by the President and Secretary of the Board and attested by the seal of the Board.

"(Note.)—There seems to be some misapprehension as to the object of the renewal fee, which makes an explanation necessary. The principal object of this provision is to overcome a legal difficulty in the matter of revoking and withholding licenses.

"A licensing body which is given discretionary power may refuse to exercise the authority conferred, in which event the only way to compel the exercise of that authority is by mandamus proceeding. The records of the courts show that it is only in cases where such discretionary power is manifestly abused that a mandamus will lie. On the other hand, where a license is granted, that license becomes a vested right during the time for which it is granted, the exercise of which right even the licensing body cannot take away except by due process of law. This has been proven by the experience of our State

Board of Health to be burdensome, expensive and impracticable; hence the necessity not only for a law which operates as a *check* upon disreputables obtaining a license, but also to provide some practical means for *taking away* that license for a good cause when it has been granted. This feature of the law restores to the licensing body, once a year, that discretionary power which seems to be the only practicable way to reach this class, who make the enactment of such laws necessary. In other words, we place them on the offensive instead of, as now, on the defensive, which we are informed upon the very best legal authority, will make practicable the suppression of the most obnoxious features of quackery and will enable the Board to exclude from the privileges of practice the disreputables already in the State. In addition, the license fee is the contribution of each reputable physician in the State to the treasury of the Board of Examiners to enable it to make a successful fight against the quacks and should not be considered as in any sense a tax, but rather as an insurance premium against fraud. Futhermore, the fees charged for the licensing of new practitioners are not sufficient to support the Board, and in order to secure the adoption of a law of this kind it will be necessary to show the legislature that it will not carry with it any appropriation from the State treasury. The failure to make a provision of this kind is the principal element of weakness of the New York law, which otherwise works well. The committee on Medical Legislation in the New York State Society, in their report last year, had this to say:

" 'Your committee take this occasion to refer to the defect in the medical practice law which makes no adequate provision for the revocation of licenses of physicians for cause. This defect we believe should be corrected, as unfortunately some regularly licensed physicians subsequently became notoriously irregular and offensive as to their methods in practice, but are still under the protection of their legally obtained license. Unjust advantage may be taken in this manner by unscrupulous physicians, and it has come to the knowledge of this committee that in other states which are infested by this class a strong movement is on foot to provide for revocation in such cases;' "

"In adopting the license feature we are following the precedent established by the Pharmacy law. When this law was first proposed it was violently opposed by a large majority of the pharmacists, but it has worked so well that its repeal would be as strenuously opposed. We believe it will prove even more satisfactory to the medical profession. In fact, we regard this provision as the strength of the bill.

"Sec. 8. The Board may refuse to issue any license provided for by this act, whether an original or renewal license, if it shall appear that the person applying therefor has been guilty of persistent inebrity, or the practice of criminal abortion, has been convicted of a crime involving moral turpitude, or has, by false or fraudulent representations, obtained practice in his or her profession, or by false or fraudulent practice of his or her profession has obtained money or any other thing of value: [NOTE. This is as near as we can get to the advertising quack by a direct legal enactment:] Provided, however, no person shall be finally refused a license until after the applicant for the same has been given at least thirty days' notice of the grounds for such refusal, and opportunity given the accused for a hearing before the Board to show cause why the Board shall not finally refuse his or her license, at which hearing the accused shall be entitled to be represented by counsel.

"SEC. 9. That any person shall be regarded as practicing medicine within the meaning of this act who shall operate for or upon, prescribe for, or otherwise treat, or profess to heal or cure any physical or mental ailment, or any physical injury to or deformatory of another: Provided, nothing in this act shall be construed to apply to the practice of dentistry or pharmacy, to the administration of domestic or family remedies in cases of emergency, to commissioned surgeons of the United States Army or Navy, or Marine Hospital Service, in the discharge of their official duties.

"SEC. 10. That any itinerant vender of any drug, nostrum, ointment or appliance of any kind intended for the treatment of disease or injury who shall vend or sell any such drug, nostrum or appliance, or who shall, by writing or printing, or any other method, profess to the public to cure or treat disease or deformity by any drug, nostrum or appliance, shall pay a license of one hundred (100) dollars per month into the treasury of the Board, to be collected by the Board in the name of the people of the State of Illinois, for the use of said Board. And it shall be lawful for the State Board of Medical Examiners to issue such license on application made to said Board, said license to be signed by the President of the Board and attested by the Secretary with the seal of the Board; but said Board may, for sufficient cause, refuse said license. Any such itinerant vender who shall vend or sell any such drug, nostrum, ointment or appliance, or who shall, by writing or printing, or any other method, profess to cure or treat diseases or deformity by any drug, nostrum or appliance, without a license so to do, shall be deemed guilty of a misdemeanor and upon conviction thereof shall be punished for the first offense by a fine of not less than one hundred dollars, or by imprisonment in the county jail for a period of not less than thirty nor more than ninety days, or by both fine and imprisonment, and for each subsequent offense the penalty shall be double that of the preceding one.

(NOTE.) This section is substantially as present law.

"SEC. 11. That the office expenses of the Board, and all expenses arising from the proper enforcement of the provisions of this act, shall be paid from the fees above provided for, and from such fines as may accrue to the Board for the violation of the provisions of this act.

"SEC. 12 That the salary of the Secretary of the Board shall be fixed by the members of the Board, and shall not exceed the sum of $3,000 per annum The members of the Board shall receive no compensation, but their traveling and other expenses incurred while employed on the business of the Board shall be paid.

"SEC. 13. That any person hereafter practicing or attempting to practice medicine or midwifery in this State without having a license so to do, as herein provided for, or contrary to the provisions of this act, shall be deemed guilty of a misdemeanor and upon conviction thereof, shall be punished for the first offense by a fine of not less than one hundred dollars and not exceeding two hundred dollars, or by imprisonment in the county jail for a period of not less than thirty days nor more than ninety days, or by both fine and imprisonment, and for each subsequent offense the penalty shall be double that of the preceding one: Provided, that the provisions of this section shall not apply for a period of six months after the appointment and organization of the first Board, to those persons who are qualified to practice medicine and surgery or midwifery, under the laws of this State in force at the time of the passage of this act.

"SEC. 14. That all suits for the recovery of the several penalties prescribed in this act shall be prosecuted in the name of "The People of the State of Illinois," in any court having jurisdiction, and it shall be the duty of the State's Attorney of the county where such offense is committed to prosecute all persons violating the provisions of this act, upon proper complaint being made. All penalties shall be paid into the treasury of the State Board of Medical Examiners.

"SEC. 15. All acts and parts of acts inconsistent or in conflict with the provisions of this act are hereby repealed."

OUR CORRESPONDENT, who is a prominent and well known member of the State Medical Association, in sending the above, writes:

*Editor Texas Medical Journal:*

"I believe, now that the election is over, that if a committee of physicians, known to him as residents of his county, would go to each representative elect in each county in the State, and in a business way lay before him their wishes, there would be but one thing else necessary to be done, and that comes first; that is, to agree among ourselves as to *what we want* and then ask for it, and if it is just and reasonable we will get it.

"I am not in favor of the three headed monstrosity proposed by the Association and shall warn our representative against its objectional feature, but advise him to compromise if need be; but whatever he does to give his support to a State Examing Board.

"With some minor changes the Illinois bill is a much better bill than the one promulgated by the Association. Now, if this is acceptable to you, how can we get it before the profession and through them to the legislators? I believe you are opposed to the recognition of the homeopaths. Do you not recognize the fact that we will not succeed without recognizing them? Shall we not have one member from the two "outs" on the board? I want to hear from you at your pleasure on this proposed bill. This bill proposes to be self-sustaining, or the Board rather; I mean it will not cost the State a cent to sustain it. All of this three board and President of State University Board and Secretary of State foolishness, as proposed by the other bill is a——————

"If you fall in with this Illinois bill and open the Red Back's broadside on them the three pounder will fail to connect, I hope."

That's it; "let us agree upon what we want, and ask for it." The profession is divided on the subject. We must respectfully decline to "open our broadside" against the Association bill; we have had our say on the subject, and if the State Medical Association desires to form an alliance with the homos and eclectics, we will no longer oppose it. We give the bill for the information of our readers.

